# A diagnostic approach to the mediastinal masses

**DOI:** 10.1007/s13244-012-0201-0

**Published:** 2012-12-06

**Authors:** Sergi Juanpere, Noemí Cañete, Pedro Ortuño, Sandra Martínez, Gloria Sanchez, Lluis Bernado

**Affiliations:** 1 Department of Diagnostic Radiology, Hospital Sant Pau i Santa Tecla, 14 Rambla Vella, 43003 Tarragona, Spain; 2 Department of Diagnostic Radiology, Dr. Josep Trueta University Hospital, Girona, Spain; 3 Department of Thoracic Surgery, Guy’s Hospital, London, UK; 4 Department of Anatomical Pathology, Dr. Josep Trueta University Hospital, Girona, Spain

**Keywords:** Mediastinum, Multidetector computed tomography, Magnetic resonance imaging, Thymoma, Cysts

## Abstract

**Background:**

Multiple different types of mediastinal masses may be encountered on imaging techniques in symptomatic or asymptomatic patients. The location and composition of these lesions are critical to narrowing the differential diagnosis.

**Methods:**

Radiological compartmentalisation of the mediastinum helps in focusing the diagnosis of masses on the basis of their site. Some diseases, however, do not occur exclusively in any specific compartment and can spread from one compartment to another.

**Results:**

Tissular components of the mass, the degree of vascularisation and the relationships with mediastinal structures assessed by computed tomography (CT) or magnetic resonance imaging (MRI) are a leading edge of the radiological diagnosis. Special applications at MRI have been developed over the recent years in order to identify accurately tissular components of the mediastinal masses. The likelihood of malignancy of the mediastinal masses is influenced by the symptomatology and the age of the patient. This article reviews the most commonly encountered mediastinal masses considering clinical history and manifestations, anatomical position and certain details seen on different imaging modalities that allow correct diagnosis in many cases.

**Conclusion:**

Familiarity with the radiological features of mediastinal masses facilitates accurate diagnosis, differentiation from other mediastinic processes and, thus, optimal patient treatment.

**Teaching Points:**

*• CT and MRI are important for the diagnosis of mediastinal masses.*

*• The location and tissue characteristics on imaging studies are critical to narrow down the differential diagnosis of mediastinal masses.*

*• Symptomatology and patient age affect the likelihood of malignancy.*

## Introduction

Mediastinal masses span a wide histopathological and radiological spectrum. The most frequent lesions encountered in the mediastinum are thymoma, neurogenic tumours and benign cysts, altogether representing 60% of patients with mediastinal masses [1]. Neurogenic tumours, germ cell neoplasms and foregut cysts represent 80% of childhood lesions, whereas primary thymic neoplasms, thyroid masses and lymphomas are the most common in adults [1].

The mediastinum is demarcated by the pleural cavities laterally, the thoracic inlet superiorly and the diaphragm inferiorly. It is further divided into anterior, middle and posterior compartments by many anatomists [2]. Anterior mediastinal tumours account for 50% of all mediastinal masses, including thymoma, teratoma, thyroid disease and lymphoma [3]. Masses of the middle mediastinum are typically congenital cysts while those arising in the posterior mediastinum are often neurogenic tumours [4].

Usual symptoms at presentation are cough, chest pain, fever/chills and dyspnea. Localising symptoms are secondary to tumour invasion (respiratory compromise; paralysis of the limbs, diaphragm and vocal cords; Horner syndrome; superior vena cava syndrome), while systemic symptoms are typically due to the release of excess hormones, antibodies or cytokines.

## Imaging modalities

**Fig. 1:**
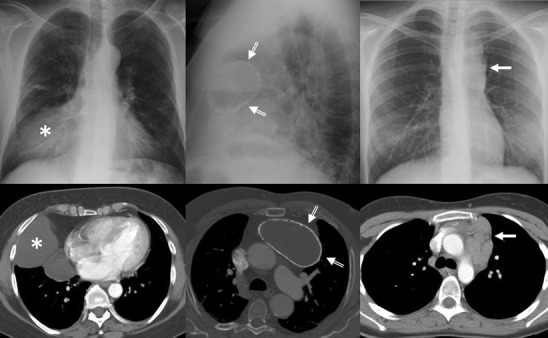
Conventional radiograph can provide information pertaining to the size, anatomical location and density of a central mass. *Right*: Frontal chest radiograph shows a sharply defined area of increased opacity with a loss of the cardiac silhouette at the border of the right side of the heart (*). Contrast-enhanced CT scan reveals a thin-walled water-attenuation lesion (*) in the right cardiophrenic angle (pericardial cyst). *Middle*: Lateral chest radiograph and contrast-enhanced CT scan show a unilocular, well-defined and homogeneously hypodense mass in the anterior mediastinum with peripheral calcification (*open arrows*) (thymic cyst). *Left*: Chest radiograph shows the aortopulmonary window with an abnormal convex border (*arrow*). Contrast-enhanced CT scan demonstrates a multilobulated mass in the anterior mediastinum (*arrow*), which accounts for the distortion of the AP window (nodular sclerosis Hodgkin disease)

Many mediastinal reflections can be appreciated at conventional radiography (CR), and their presence or distortion is the key to the interpretation of mediastinal abnormalities [2]. However, computed tomography (CT) is the most important tool in the evaluation of a mediastinal mass [5]. Characterisation on CT is based on specific attenuation of air, fat, water and calcium (Fig. 1). High-resolution multiplanar reformation images display the detailed anatomical relationship of the tumour with the adjacent structures. An excellent soft tissue contrast also designates magnetic resonance imaging (MRI) as an ideal tool to evaluate tumours of the mediastinum [6]. Assessment of preoperative relationships with the pericardium, heart cavities, spinal cord and vascular involvement is a common indication. Chemical-shift MRI has been shown to be useful in distinguishing normal thymus and thymic hyperplasia from thymic neoplasms and lymphoma [7]. Diffusion-weighted MRI (DWI) is another special application that unveils minute metabolic and biophysical differences between tissues. According to Gumustas et al. [8], the mean apparent diffusion coefficient for the malignant mediastinal entities could be significantly lower than that for the benign diseases.

## Mediastinal masses

**Fig. 2:**
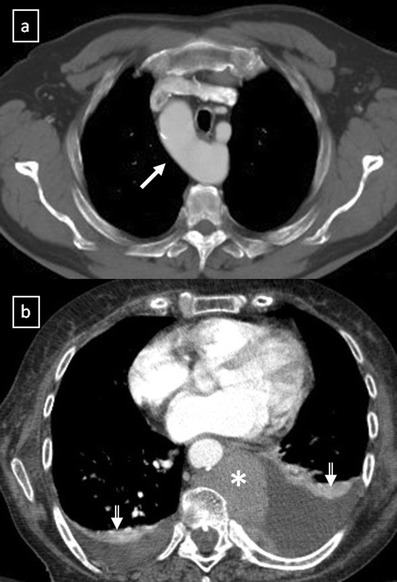
**a** Contrast-enhanced CT scan shows a right-sided aortic arch (*arrow*) in an asymptomatic man with an absence of the aortic knuckle on the left. **b** Contrast-enhanced CT scan demonstrates a soft tissue posterior mediastinal mass (*) in a 66-year-old woman with multiple myeloma diagnosis. Note the mass effect to the descending aorta and left auricula by the mass and the bilateral pleural effusion (*open arrows*)

**Fig. 3:**
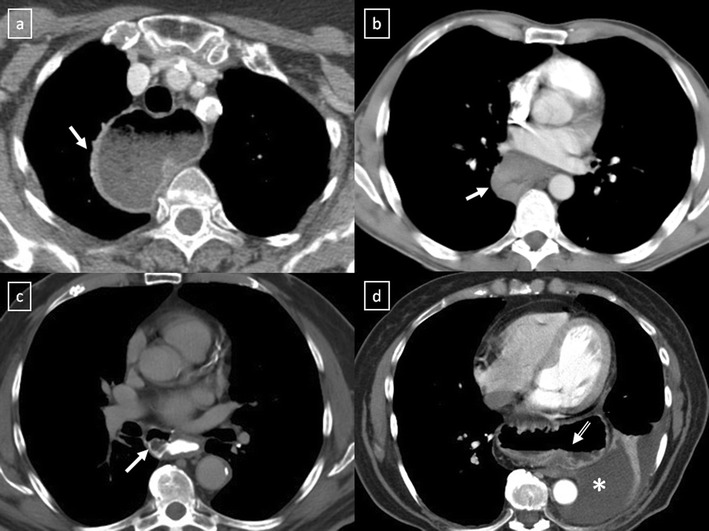
Different masses arising from the digestive tract. **a** Oesophageal stenosis after Nissen fundoplication (*arrow*). **b** Posterior mediastinal mass (*arrow*) in relation with squamous-cell carcinoma of the oesophagus. **c** Oesophageal diverticulum (*arrow*) in a patient with oesophageal achalasia. Note the thickened oesophagus. **d** Hiatal hernia is a frequent incidental finding with or without air or air-fluid level (*open arrow*). * Pleural effusion

Many entities that involve the mediastinum correspond to anatomical variants or masses arising from the spine or from the digestive tract, and should not be considered true mediastinal masses (Figs. 2 and 3). Lymph node enlargement represents a frequent cause of mediastinal masses [9].

### Fatty masses

*Lipomas* predominantly occur in the anterior mediastinum and are reported to represent 1.6–2.3% of all primary mediastinal tumours [10]. At CT, lipomas have homogeneous fat attenuation of approximately -100 HU. *Liposarcoma* frequently occurs in the posterior mediastinum and it is usually symptomatic at the time of presentation, in contrast to lipoma [11]. Inhomogeneous appearance on CT or MRI differentiates liposarcoma from lipoma [12].

**Fig. 4:**
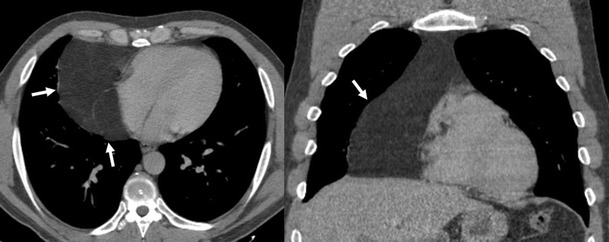
Thymolipoma in a 47-year-old asymptomatic man. Axial and coronal multiplanar reconstruction of non-contrast-enhanced CT scan show a large and well-defined mass (*arrows*) that has extensive fat content and contains small amounts of thin fibrous septa

*Thymolipoma* is a rare, benign, well-encapsulated thymic tumour that accounts for about 2–9% of thymic neoplasms [10, 12–14]. Tumours occur most frequently in the cardiophrenic angle of asymptomatic young adults without sex predilection. The fat content usually constitutes 50–85% of the lesion but has been reported to account for as much as 95% of the tumour [10]. Associations with myasthenia gravis, Graves disease and haematological disorders have been reported [14]. At CR, thymolipomas may mimic cardiomegaly, excessive epicardial fat, diaphragmatic elevation, lobar collapse or a pericardial cyst. CT or MRI is required to establish the diagnosis by showing a well-defined encapsulated mass that has extensive fat content and contains small amounts of solid areas and fibrous septa [12] (Fig. 4).

### Cystic masses

**Fig. 5:**
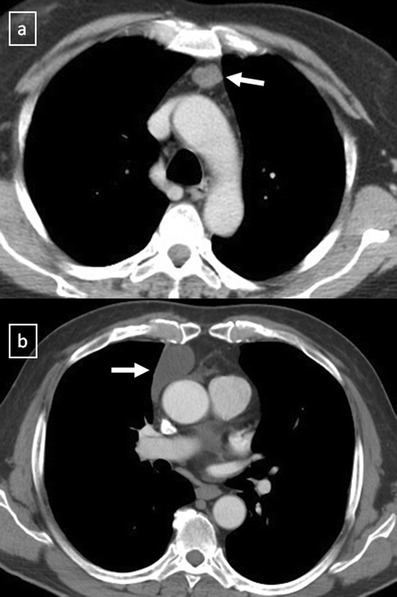
A well-marginated mass with a homogeneous attenuation, usually in the range of water attenuation (0–20 HU) and without an enhancement of the wall or infiltrative appearance are the typical features of benign mediastinal cysts. Probably thymic cystic (**a**) and pericardial cyst (**b**)

**Fig. 6:**
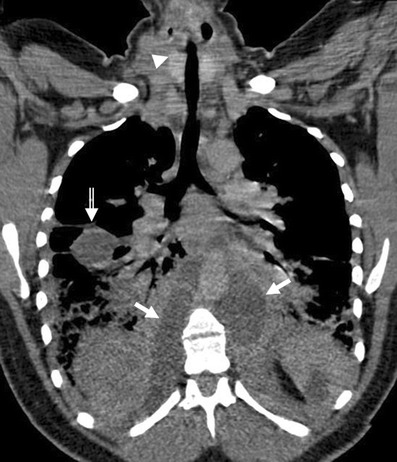
Oblique-coronal multiplanar reconstruction of contrast-enhanced CT scan of a 33-year-old woman with a descending necrotising mediastinitis. Hypodense para-aortic areas correspond with fluid-collections (*arrows*) that extend to the retroperitoneum. Pleural effusion in the fissure is also be seen (*open arrow*). Note the visceral space involvement (*arrowhead*)

Mediastinal primary cysts represent 15–20% of all primary mediastinal masses [1, 15]. A smooth or oval mass with a homogeneous attenuation, with no enhancement of cyst contents and no infiltration of adjacent structures are the usual CT features of benign mediastinal cyst (Fig. 5). Any cyst may have a higher attenuation due to its calcic, proteinaceous, mucous or haemorrhagic content. Cysts typically show high signal intensity on T2-weighted MR images. True cystic lesions should be differentiated from the cystic degenerative changes observed in many solid tumours, in lymphomas before or after treatment, or in abscesses (Fig. 6).

**Fig. 7:**
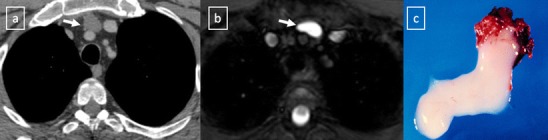
Bronchogenic cyst in a 37-year-old man. **a** Non-contrast-enhanced CT scan shows a homogeneous anterior mediastinal mass with smooth contours and oval shape (*arrow*). The mass is isodense relative to chest wall muscle. **b** T2-weighted MR image shows the same mass (*arrow*) with markedly high signal intensity. **c** Although its localisation, a bronchogenic cyst was confirmed by histological examination after surgical resection

*Bronchogenic cysts* result from abnormal ventral budding or branching of the tracheobronchial tree during embryologic development [15–17]. They are lined by respiratory epithelium and their capsule contains cartilage, smooth muscle and mucous gland tissue. They are stable in size, except when complicated by infection or haemorrhage. Approximately 40% of bronchogenic cysts are symptomatic, resulting in cough, dyspnea or chest pain [4]. The bronchogenic cyst is commonly located in the near carina (52%) and in the paratracheal region (19%) [5]. The anterior mediastinum is a rare location of the bronchogenic cyst [17] (Fig. 7). Air within the cyst is suggestive of secondary infection and communication with the tracheobronchial tree.

**Fig. 8:**
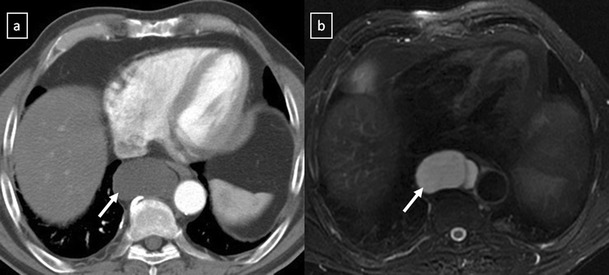
Duplication cyst in a 42-year-old asymptomatic man. **a** Contrast-enhanced CT scan shows a round well marginated mass (*arrow*) adjacent to the oesophagus with homogeneous water-attenuation. **b** The lesion (*arrow*) presents a bright signal intensity on T2-weighted fat-suppressed MR image

*Duplication cysts* are uncommon lesions lined by gastrointestinal tract mucosa and generally asymptomatic. However, if they contain gastric or pancreatic mucosa, there is the added risk of haemorrhage or rupture of the cyst from mucosal secretions. The majority of them are detected adjacent to or within the oesophageal wall (Fig. 8). Duplication cysts are indistinguishable from bronchogenic cysts on CT and MRI.

*Mediastinal neuroenteric cysts* are anomalous protrusions of the leptomeninges through intervertebral foramen or defects in the vertebral body. They are associated with multiple vertebral anomalies and with neurofibromatosis [15, 16].

**Fig. 9:**
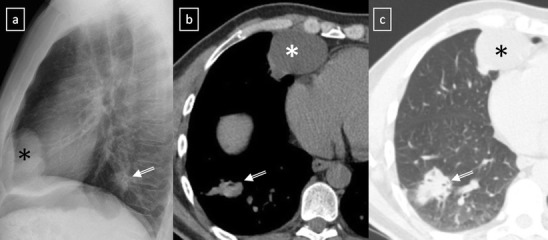
**a** Lateral chest radiograph of a 58-year-old smoker man allergic to iodine shows a well-defined mass (*) in the cardiophrenic space and a nodular lung opacity (*open arrow*) in the lower lung parenchyma. **b**, **c** Non-contrast-enhanced CT scan confirms the presence of a fluid-attenuation mass (*) in the right cardiophrenic angle (pericardial cyst) and demonstrates a suspicious lung opacity (*open arrows*) in the right lower lobe of the lung (squamous-cell carcinoma)

*Pericardial cyst* is a benign lesion accounting for 5–10% of all mediastinal tumours [11]. Most pericardial cysts are unilocular and commonly located in the right cardiophrenic space (Fig. 9). However, they may occur anywhere in relation to the pericardium.

**Fig. 10:**
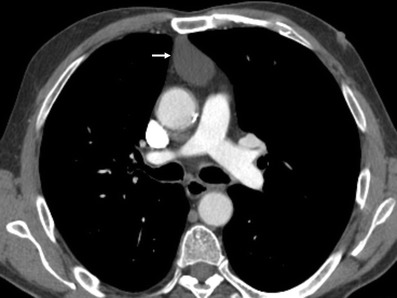
Congenital thymic cyst in a 47-year-old man. Contrast-enhanced CT scan demonstrates a unilocular unenhanced lesion in the anterior mediastinum which shows a homogeneous fluid-attenuation (*arrow*)

**Fig. 11:**
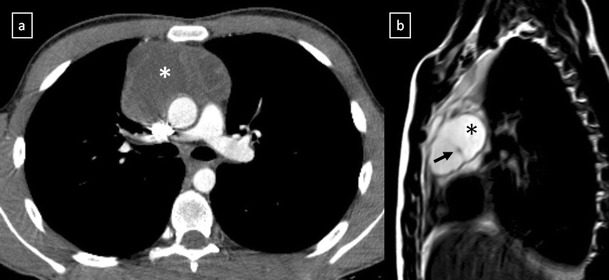
Acquired thymic cyst in a 43-year-old man. **a** Contrast-enhanced CT scan shows a well-defined water-attenuation multiloculated mass (*) in the anterior mediastinum. **b** Sagittal T2-weighted MR sequence demonstrates a multiloculated mass with typical high signal intensitiy and fine internal septa within (*arrow*)

*Thymic cyst* represents 1% of all mediastinal masses [15]. Congenital cysts derive from remnants of the thymopharyngeal duct, they are typically unilocular and contain clear fluid (Fig. 10). In contrast, acquired thymic cysts are much more common, tend to be multilocular (Fig. 11) and may arise in association with neoplasms such as thymomas, lymphomas or germ cell tumours. Thymic cysts may also be seen in the anterior mediastinum after radiation therapy of Hodgkin’s disease, after inflammatory processes and occasionally in patients with AIDS, particularly in children [18].

**Fig. 12:**
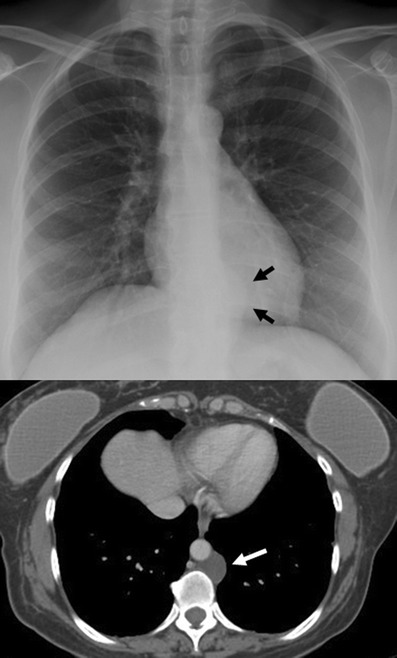
A cystic lymphangioma (also referred to as hygroma) in a 47-year-old woman with retroperitoneal disease. A posterior mediastinal mass with a homogeneous fluid-attenuation is identified on CT (*white arrow*) and on posteroanterior chest radiograph as a mass disrupting left paraspinal line inferiorly (*black arrows*)

*Lymphangioma* is a rare benign lesion of lymphatic origin that represents 0.7–4.5% of all mediastinal tumours in adult population [15]. Lymphangiomas involve the neck or the axillary region in more than 80% of the cases and the thorax in 10% of the cases [19] (Fig. 12).

**Fig. 13:**
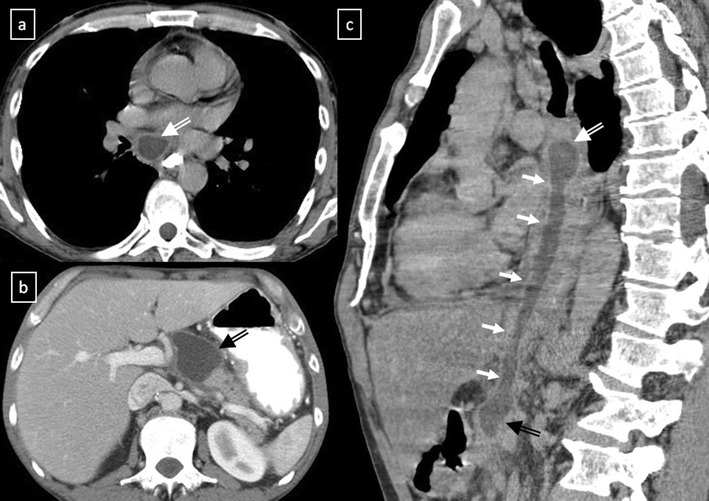
Pancreatic pseudocyst in a 52-year-old man with a recurrent pancreatitis history. Axial (**a**, **b**) and oblique-sagittal multiplanar reconstruction (**c**) CT scan show a thin-walled peri-oesophageal fluid-lesion (*white open arrows*) that comunicates with an intrapancreatic fluid-collection (*black open arrows*) through oesophageal hiatus by a duct fistula (*white arrows*)

*Pancreatic pseudocyst* can extend into the mediastinum via the oesophageal or aortic hiatus. CT shows a thin-walled, fluid-containing cyst within the posterior mediastinum which may be in continuity with the intrapancreatic or peripancreatic fluid collections (Fig. 13).

### Solid masses

**Fig. 14:**
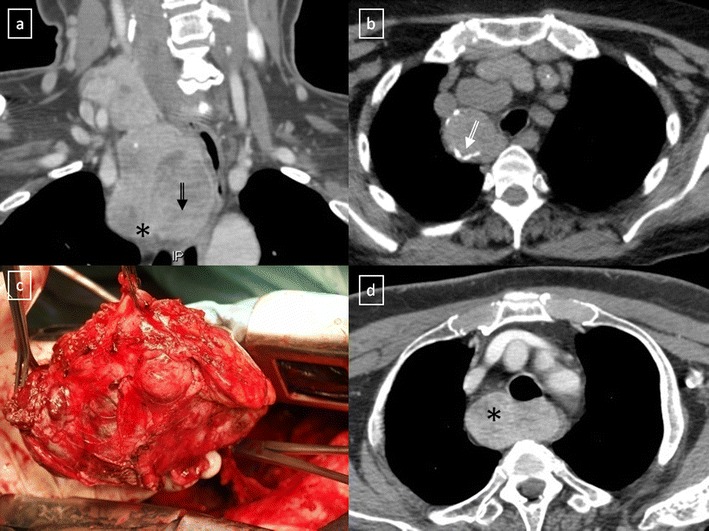
Mediastinal goitres. **a** Coronal multiplanar reconstruction CT scan demonstrates an anterior mediastinal mass (*) arising from the thyroid more superiorly. Note the cystic degeneration within goitre (*open arrow*). **b** Non-contrast-enhanced CT scan shows a unilateral mediastinal goitre with peripheral areas of calcification (*open arrow*). **c** Photograph of the resected surgical specimen shows a lobulated and heterogeneous appearance of the mass. **d** Contrast-enhanced CT scan shows a well marginated posterior mediastinal goitre (*) with marked contrast enhancement

**Fig. 15:**
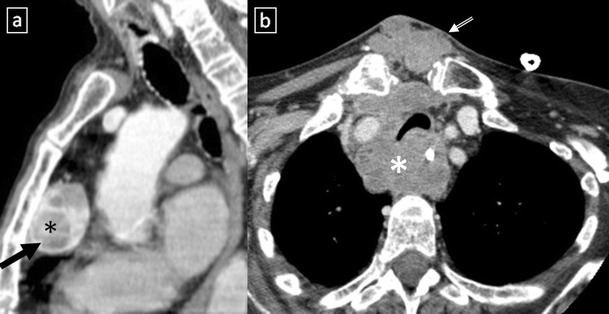
**a** Heterotopic mediastinal goitre. Sagittal contrast-enhanced CT scan demonstrates a well-defined mass (*arrow*) located in the retrosternal space which shows an intense and heterogeneous contrast enhancement due to the presence of cystic areas (*). No connection to the thyroid gland from the neck. **b** Anaplastic thyroid carcinoma in a 64-year-old woman with respiratory failure. Contrast-enhanced CT scan shows an anterior mediastinal soft tissue mass (*) which surrounds great vessels and oesophagus and compresses trachea. Note the extension to the suprasternal fossa (*open arrow*)

*Mediastinal goitre* generally represents direct contiguous growth of a goitre into the anterior or superior mediastinum. Typical features of mediastinal goitres are encapsulated and lobulated mass with inhomogeneous appearance with cystic areas, calcifications and marked contrast enhancement. (Fig. 14). An intrathoracic thyroid mass developing from heterotopic thyroid tissue without any connection to the thyroid in the neck is extremely rare (Fig. 15a). The presence of ill-defined margins, invasion of adjacent structures and nearby lymph node enlargement suggests the diagnosis of thyroid cancer [20] (Fig. 15b).

**Fig. 16:**
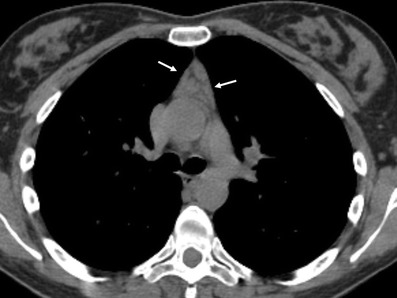
Thymic lymphoid hyperplasia in a 41-year-old woman with clinical diagnosis of myasthenia gravis. Non-contrast-enhanced CT scan shows an enlarged thymic gland (*arrows*) without mass effect on adjacent structures

*Thymic hyperplasia* can be divided into two distinct histological types [13, 14]. *True thymic hyperplasia* is defined as enlargement of the thymus, which generally retains its normal shape. This disease entity is observed when a patient is recovering from some recent stress (such as chemotherapy, corticosteroid therapy, irradiation or thermal burns). The phenomenon known as rebound hypeplasia is defined as a greater than 50% increase in thymic volume over baseline after such stress [14]. Among patients who undergo chemotherapy, approximately 10–25% may develop rebound hyperplasia [13]. *Thymic lymphoid (follicular) hyperplasia* of the thymus refers to the presence of an increased number of lymphoid follicles. It is commonly associated with autoimmune diseases, being seen in up to 65% of cases with myasthenia gravis [14], and it has been reported to occur in the early stages of human immunodeficiency virus infection. At CT it may appear normal (45%), enlarged (35%) (Fig. 16) or as a focal thymic mass (20%) [13].

**Fig. 17:**
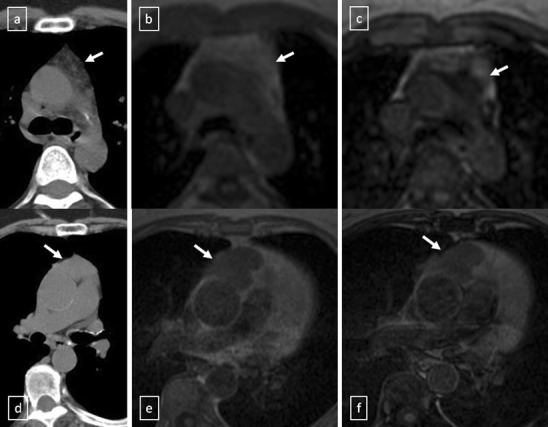
*a–c* Thymic hyperplasia in a 43-year-old woman (*arrows*). **a** Non-contrast-enhanced CT scan reveals a lobulated lesion with smooth margins in anterior mediastinum. **b** Lesion appears slightly hyperintense on in-phase gradient-echo T1-weighted MR image. **c** Opposed-phase gradient-echo T1-weighted MR image shows decreased signal intensity within the lesion, confirming presence of fat. *d–f* Stage II thymoma (WHO type B2) in a 62-year-old woman (*arrows*). **d** Non-contrast-enhanced CT scan shows an anterior mediastinal soft tissue mass. **e** The lesion shows an intermediate signal intensity on in-phase gradient-echo T1-weighted MR image. **f** There is no substantial decrease in signal intensity relative to in-phase MR image on opposed-phase sequence

It is important for radiologists to be able to distinguish thymic hyperplasia from neoplasm. Diffuse symmetric enlargement of the gland, a smooth contour and normal vessels are the key morphological features of hyperplasia, whereas neoplasm tends to manifest as a focal mass with nodular contour and necrotic or calcified foci. Detecting fat in the thymus is particularly relevant in these situations. According to Takahashi et al. [21], chemical-shift MRI can be useful in this situation. Thymic hyperplasia reveals a relative signal loss on opposed-phase chemical-shift MRI that it is different from no significant signal change between in-phase and opposed-phase chemical-shift MR images in patients with malignancy (Fig. 17).

*Thymoma* is the most common primary neoplasm of the anterior mediastinum but accounts for less than 1% of all adult malignancies [22, 23]. Thymomas typically occur in patients older than 40 years of age, being rare in children, and affecting men and women equally [23]. Between 20% and 30% of patients with thymoma have pressure-induced symptoms [13]. Myasthenia gravis associated with thymoma occurs most frequently in women. Between 30% and 50% of patients with a thymoma have myasthenia gravis, whereas 10–15% of patients with myasthenia gravis have a thymoma.

**Fig. 18:**
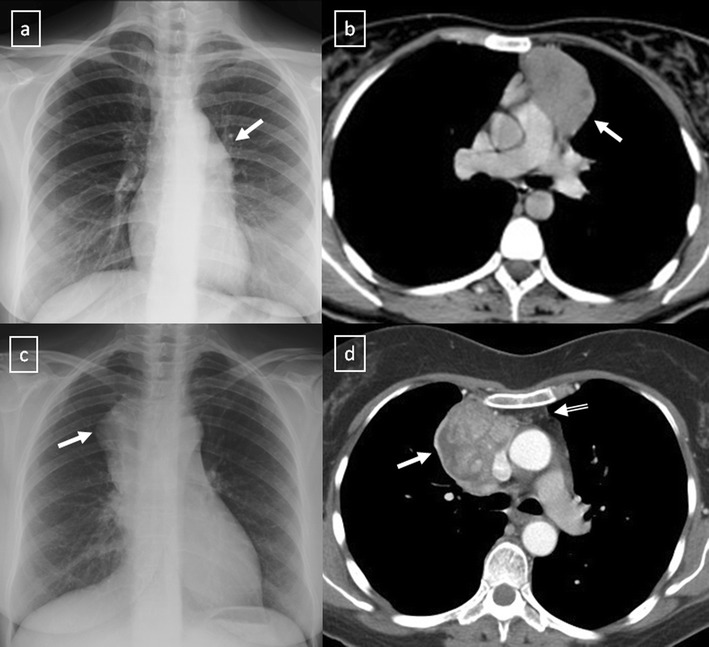
**a**, **b** Stage II thymoma (WHO type B1) in a 33-year-old woman who presented with myasthenia gravis. Frontal chest radiograph shows a hilum overlay sign (*arrow*) of a suggestive anterior mediastinal mass. Contrast-enhanced CT scan confirms the presence of a low-heterogeneous anterior mediastinal mass (*arrow*). Note the indentation of the arterial trunk pulmonary by the mass. **c**, **d** Stage III thymoma (WHO type B2) in a 54-year-old woman. Frontal chest radiograph reveals a lobulated mediastinal mass (*arrow*) on the right side. Contrast-enhanced CT scan demonstrates an enhanced anterior mediastinal mass (*arrow*) with infiltration of surrounding fat (*open arrow*)

**Fig. 19:**
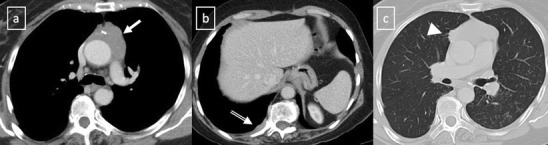
CT is the imaging modality of choice for evaluating staging thymoma. Stage IVa thymoma (WHO type B3) in a 69-year-old woman. **a**, **b** Contrast-enhanced CT scan shows a well-circumscribed, flattened soft tissue lesion in the anterior mediastinum with calcification (*arrow*). Note the lobulated contour of the mass and the loss of the fat plane between the mass and the aorta. Pleural seeding is identified as an enhancing pleura-based nodule (*open arrow*). **c** Irregular border between the mediastinal mass and the lung parenchyma (*arrowhead*) is observed as a sign of locally advanced disease. Note the cellular bronchiolitis in the left lower lobe

**Fig. 20:**
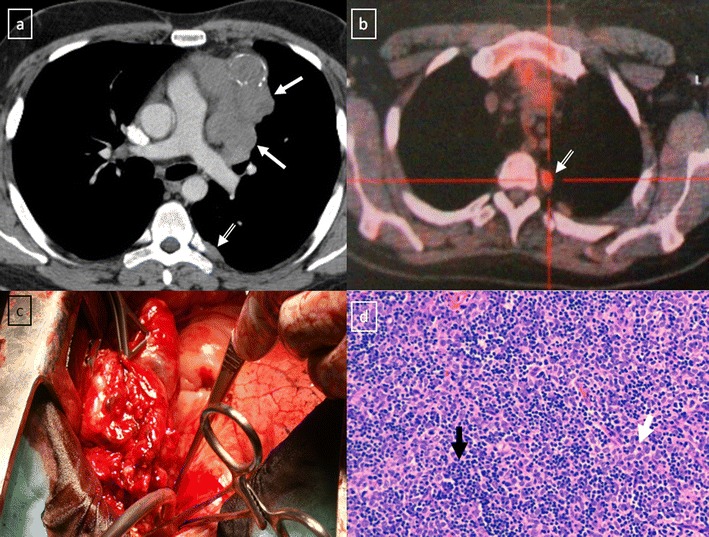
Stage IVa thymoma (WHO type B2) in a 46-year-old man. **a** Contrast-enhanced CT scan reveals an anterior mediastinal mass (*arrows*) with irregular contours, homogeneous enhancement and peripheral and central calcification as well as a pleural nodule (*open arrow*). **b** On an axial FDG-positron emission tomography (PET) image, the pleural nodule is FDG avid, confirming a drop metastasis. **c** Image during the surgical resection. **d** Photomicrograph (haematoxylin-eosin stain) of tissue from the lesion shows roughly equal numbers of epithelial cells (*white arrow*) and lymphocytes (*black arrow*) corresponding thymoma WHO type B2

**Fig. 21:**
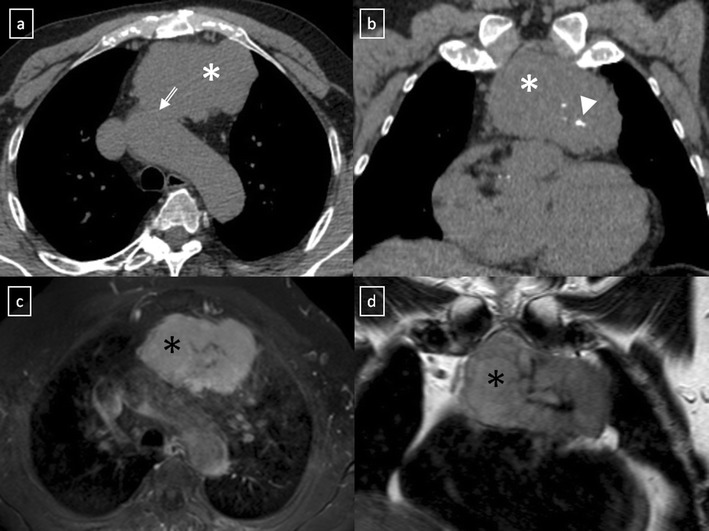
Axial (**a**) and coronal multiplanar reconstruction (**b**) of a non-contrast-enhanced CT scan of a 57-year-old man allergic to iodine with a thymoma. A solid lobulated thymic mass (*) with clumps of calcifications within (*arrowhead*) is identified. Note the absence of a fat plane between the tumour and the aorta (*open arrow*). **d** Coronal T2-weighted MR image shows a typical signal hyperintensity of the tumour lesion (*). **c** Axial contrast-enhanced fat-suppressed T1-weighted MR image reveals a homogeneously enhanced solid tumour (*) which arises from the thymus. Although MRI demonstrates the presence of fat cleavage plane between ascending aorta and the tumour, a thymoma (WHO type A) with microscopic transcapsular invasion (Masaoka stage II) was confirmed after surgical resection

The updated histological classification elaborated by WHO in 2004 classified different types of thymomas (Table 1) on the basis of the morphology of the neoplastic epithelial cells together with the lymphocyte-epithelial cell ratio [23, 24]. In contrast to histological classification, the stage of thymoma has clinical implications and it is a useful tool for management decisions. The Masaoka-Koga staging system is the most commonly used and describes thymomas in terms of the local extension of the tumour [14, 23–26] (Table 2). The Masaoka staging system is one of the two factors, including completeness of surgical resection, that most strongly correlates with prognosis of thymomas [23]. The role of imaging is to initially diagnose and properly stage thymoma, with emphasis on the detection of local invasion and distant spread of disease. Between 45 and 80% of thymomas are visible by chest radiography [22]. On CT scans, thymomas usually appear as homogeneous solid masses with soft-tissue attenuation and well-demarcated borders, located anywhere from the thoracic inlet to the cardiophrenic angle. Thymomas may be oval, round or lobulated and when they are large, cystic or necrotic degeneration may be shown (Fig. 18). Calcification may be present in the capsule or throughout the mass. Certain findings, such as encasement of mediastinal structures, infiltration of fat planes, irregular interface between the mass and lung parenchyma, and direct signs of vascular involvement are highly suggestive of invasion (Fig. 19). Pleural dissemination (“drop metastases”) manifests as one or more pleural nodules or masses, and they are almost always ipsilateral to the tumour [23] (Fig. 20). Thymoma rarely presents with metastatic lymphadenopathy, metastatic pulmonary nodules or pleural effusion. At MRI, thymomas commonly appear as homogeneous or heterogeneous masses with low to intermediate signal intensity on T1-weighted images and with high signal intensity on T2-weighted images (Fig. 21). MRI can prove useful in identifying the nodular wall thickening detected in cystic thymomas, absent from congenital cysts [22].

**Table 1:** WHO classification for thymoma

2004 WHO classification	Description
A (spindle cell thymoma; medullary thymoma)	Bland spindle/oval epithelial tumour cells with few or no lymphocytes
AB (mixed thymoma)	Mixture of a lymphocyte-poor type A thymoma component and a more lymphocyte-rich type B-like component (smaller and paler than those of B1 or B2 thymomas). Lymphocytes are more numerous than in the type A component, but may be less numerous than in B1 thymomas
B1 (lymphocyte-rich thymoma; lymphocytic thymoma; organoid thymoma; predominantly cortical thymoma)	Epithelial cells with a histological appearance practically indistinguishable from the normal thymus, composed predominantly of areas resembling cortex with epithelial cells scattered in a prominent population of immature lymphocytes
B2 (cortical thymoma)	Tumour cells closely resembling the predominant epithelial cells of the normal thymic cortex. A background population of immature T cells is always present and usually outnumbers the neoplastic epithelial cells
B3 (epithelial thymoma; squamoid thymoma)	Medium–sized round or polygonal cells with slight atypia. The epithelial cells are mixed with a minor component of intraepithelial lymphocytes

From Travis et al. [24]

**Table 2:** Masaoka-Koga staging system of thymoma

Stage	Description
I	Macroscopically and microscopically encapsulated tumour
IIa	Microscopic invasion through the capsule
IIb	Macroscopic invasion into surrounding fatty tissue
III	Invasion into a neighbouring organ such as the pericardium, great vessels or lung
IVa	Pleural or pericardial dissemination
IVb	Lymphatic-haematogenous metastases

From references [14, 23–26]

*Thymic carcinoma* accounts for about 20% of thymic epithelial tumours with a mean age of 50 years [13]. Typical appearance is a multilobulated and heterogeneous mass that may contain areas of calcification or haemorrhage. Distant metastasis are present at the initial diagnosis in 50–65% [13]. Sadohara et al. [27] found that irregular contour, necrotic or cystic components, heterogeneous enhancement, lymphadenopathy and great vessel invasion strongly favoured thymic carcinoma.

*Thymic carcinoids* are rare, well-differentiated neuroendocrine tumours, which have a male predilection of 3:1 [13, 14]. They often present with endocrine disorder. Thymic carcinoid usually manifests as a large anterior mediastinal mass often with metastases.

**Fig. 22:**
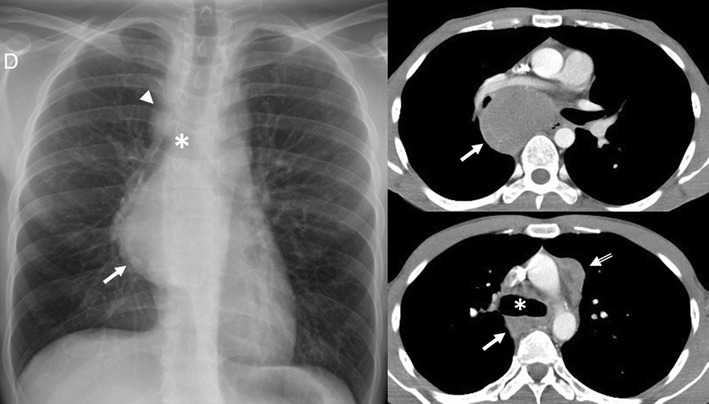
A 28-year-old man with Hodgkin lymphoma. Frontal chest radiograph and contrast-enhanced CT scan show a homogeneous soft tissue mass at the level of the subcarina (*arrows*). An aortopulmonary window lymphadenopathy can be detected on CT scan (*open arrow*). The right paratracheal stripe is not seen on frontal chest radiograph, having been most probably obliterated by a right paratracheal lymphadenopathy (*arrowhead*). * Carina

*Lymphoproliferative disorders*. Primary mediastinal lymphoma usually occurs in the anterior mediastinum. Malignant lymphoma accounts for nearly 20% of all mediastinum neoplasms in adults and 50% in children [5]. Lymphomas are the most common cause of masses in the paediatric mediastinum [18]. Hodgkin lymphoma (Fig. 22) represents approximately 50–70% of mediastinal lymphomas, while non-Hodgkin lymphoma comprises 15–25% [4]. Pleural and pericardial effusions are often common features in all types of lymphoma.

**Fig. 23:**
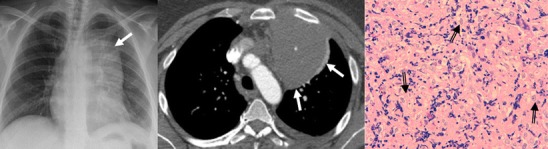
Nodular sclerosis Hodgkin lymphoma in a 44-year-old woman. Frontal chest radiograph shows a large, well-defined mediastinal mass with increased density (*arrow*). Contrast-enhanced CT scan shows a bulky soft tissue mass (*arrows*) with homogeneous CT-attenuation value occupying prevascular space. Note the left internal mammarian artery completely surrounded by the lesion. Photomicrograph reveals numerous neoplastic lacunar cells (*arrows*) in a background of small lymphocytes, histiocytes and eosinophils, which supports the diagnosis of nodular sclerosis type Hodgkin lymphoma

*Hodgkin disease* (HD) has a bimodal distribution of incidence peaking in young adulthood and again after the age of 50 years [4]. Most patients experience constitutional symptoms. Four subtypes of HD are described: nodular sclerosis (by far the most common histological subtype) (Fig. 23), lymphocyte-rich, mixed cellularity and lymphocyte depleted HD [4, 5]. CR is abnormal in up to 76% of patients with HD, often showing enlargement of the prevascular and paratracheal nodes [4]. Characteristic features on imaging are a homogeneous soft-tissue anterior mediastinal mass with mild to moderate contrast enhancement, irregular contours, surface lobulation, absence of vascular involvement, and high prevalence of associated mediastinal lymphadenopathy [3]. Cystic and necrotic changes can be identified.

**Fig. 24:**
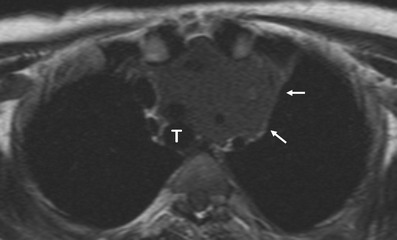
Axial T1-weighted MR image of a 16-year-old man with a solid, large mass (*arrows*) in the anterior and superior mediastinum. Supra-aortic trunks are almost completely surrounded by the lesion and trachea (*T*) is displaced to the contralateral side by the lesion. Pathological analysis demonstrated a T-cell lymphoblastic lymphoma

**Fig. 25:**
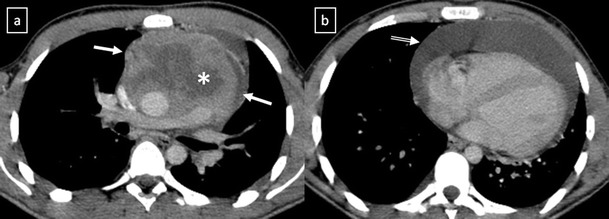
Primary mediastinal diffuse large B-cell lymphoma in a 19-year-old man. Contrast-enhanced CT scan shows a bulky soft tissue mass in the anterior mediastinum (*arrows*in **a**), which shows heterogeneous CT-attenuation values with cystic changes within (*). Pericardial effusion is also observed (*open arrow* in **b**)

The two most common forms of mediastinal *non-Hodgkin disease* (NHD) include diffuse large B-cell lymphoma and T-cell lymphoblastic lymphoma. T-cell lymphoblastic lymphoma mainly occurs in children and adolescents. The most common CT appearance is a large mediastinal mass representing thymic and lymph node enlargement, which compresses the airway and cardiovascular structures (Fig. 24). Low attenuation areas reflecting necrosis are commonly seen. Primary mediastinal diffuse large B-cell lymphomas tend to occur in young to middle-aged adults with a mean age of 30 [5]. It accounts for 7% of all cases of NHD and about 10% of all cases of high-grade NHD [28]. The tumours appear as a large, smooth or lobulated, anterior mediastinal mass in nearly all patients. On CT the tumours show low attenuation areas, representing haemorrhage, necrosis or cystic degeneration in 50% of the cases and heterogeneous enhancement in about 40% of the cases [5] (Fig. 25). Primary mediastinal B-cell lymphoma recurs in the chest. Consequently, chest CT examination alone is sufficient for routine follow-up of these patients [28].

*Germ cell tumours* (GCTs) mainly arise in gonads and in the midline of the body as well, the mediastinum being the most common extragonadal site. GCTs account for 10–15% of anterior mediastinal masses in adults and 25% in children [5]. Only 3% of them arise in the posterior mediastinum [4]. Pathological classifications include teratomas and non-teratomatous germ cell tumours.

**Fig. 26:**
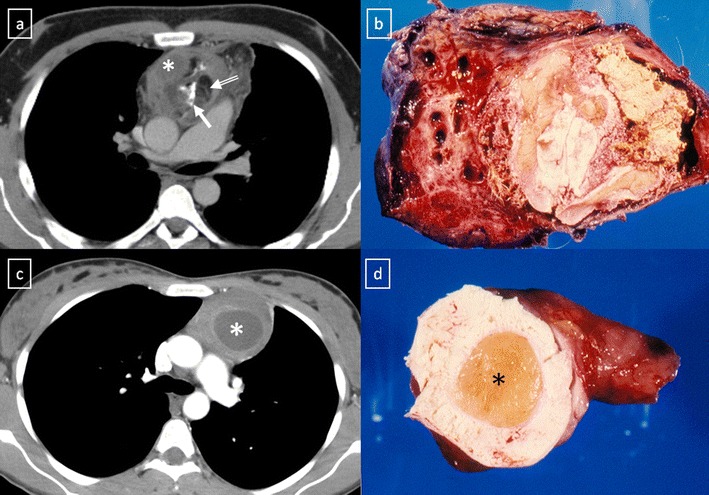
Chest imaging shows well the highly heterogeneous contents of mediastinal teratomas. **a** Mature cystic teratoma in a 40-year-old man. Contrast-enhanced CT scan shows a heterogeneous anterior mediastinal mass with areas of fat (*open arrow*), calcification (*arrow*) and fluid attenuation (*). Posterior displacement of mediastinal structures is also be seen*.* **b** Photograph of the surgical specimen. **c** Contrast-enhanced CT scan of an asymptomatic 24-year-old woman demonstrates a well-defined uniloculated mass located in prevascular space which shows a cystic changes within (*). Non foci of calcification were identified. **d** The mass was surgically removed and pathological examination confirmed a benign teratoma

*Teratoma* is the most common mediastinal GCT [4]. Mature teratomas are usually asymptomatic and represent 60–70% of all mediastinal GCTs [5]. They are composed of well-differentiated benign tissues with predominant ectodermal element. If a teratoma contains fetal tissue or neuroendocrine tissue, it is defined as immature and malignant with a poor prognosis. On CT, teratoma most commonly appears as a well-defined unilocular or multilocular cystic lesion containing fluid, soft tissue and fat attenuation (Fig. 26). Calcifications may be focal, rim-like or, in rare cases, representative of teeth or bone. Common combinations of internal components of mature teratomas include soft tissue, fluid, fat and calcification in 39%; soft tissue, fluid and fat in 24%; and soft tissue and fluid in 15%. In 15% of the cases, mature teratomas appear as non-specific cystic lesions without fat or calcium [5]. On MRI, teratomas typically demonstrate heterogeneous signal intensity, representing various internal elements. Fat-fluid levels within the lesion are virtually diagnostic of teratoma. Ruptured teratomas show an adjacent consolidation, atelectasis and pleural or pericardial effusion than do unruptured teratomas.

**Fig. 27:**
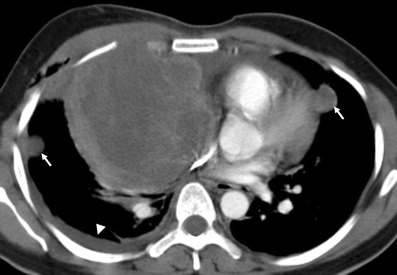
A 19-year-old man with seminoma. Contrast-enhanced axial CT scan demonstrates a large mass in the right side of the mediastinum with an obvious mass effect on great vessels and heart. The mass shows heterogeneous CT-attenuation values probably secondary to haemorrhage and coagulation necrosis. Note also a right pleural effusion (*arrowhead*) and multiple lung metastasis (*arrows*)

**Fig. 28:**
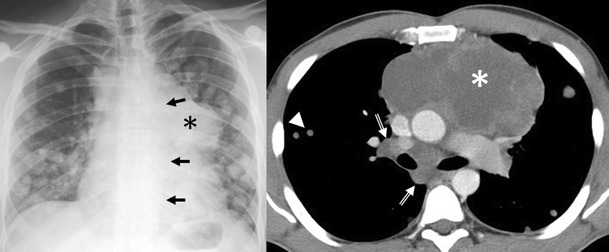
Non-seminomatous malignant germ cell tumour of the anterior mediastinum in a 25-year-old man with chest pain and high serum level of α-fetoprotein at admission (25.396 ng/ml). Frontal chest radiograph shows a central mass (*). The descending aorta is clearly seen (*arrows*), indicating that the mass is not within the posterior mediastinum. Multiple nodules in bilateral pulmonary field are also observed. A contrast-enhanced CT scan confirms a mass of low attenuation (*) in the anterior mediastinum that compresses the pulmonary artery. Bilateral lung metastasis (*arrowheads*) and hilar and subcarinal lymphadenopathy is identified (*open arrows*)

The *non-teratomatous germ cell tumours* (NTGCT) are rare and malignant tumours which usually occur in young males and most frequently affect the anterosuperior mediastinum [29]. These tumours grow rapidly and develop large, bulky, ill-circumscribed masses with lobulated shape. Primary mediastinal seminomas comprise 25–50% of malignant mediastinal GCTs [4] and occur almost exclusively in males during the period from the second to fourth decades of life [5]. At imaging, the tumours typically have homogenous appearance and show minimal contrast enhancement. Areas of degeneration due to haemorrhage and coagulation necrosis may be present (Fig. 27). Metastasis to lymph nodes and bone does occur. Non-seminomatous germ cell tumours include yolk sac tumours, endodermal sinus tumours, embryonal carcinomas, choriocarcinomas and mixed germ cell tumours, which present as large masses typically with marked heterogeneous attenuation. At diagnosis, 85% of patients are symptomatic [4]. Invasion of adjacent structures and distant metastasis may occur. Pleural and pericardial effusions are common. Measuring AFP and ß-hCG levels is important when making the diagnosis (Fig. 28).

**Fig. 29:**
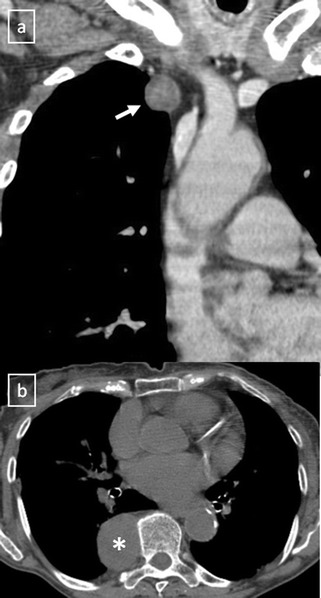
Peripheral nerve tumours usually show a markedly convex mass arising from the mediastinum. **a** Coronal multiplanar reconstruction of contrast-enhanced CT scan of an asymptomatic 59-year-old man with a mass in right superior mediastinum (*arrow*). Histological examination confirmed a schwannoma arising from the phrenic nerve. **b** Schwannoma in a 77-year-old woman. Non-contrast-enhanced CT scan shows a well-defined and homogeneous paravertebral mass (*)

*Neurogenic tumours* represent approximately 20% of all adults and 35% of all paediatric mediastinal tumours and they are the most common cause of a posterior mediastinal mass [30]. Seventy to eighty percent of neurogenic tumours are benign, and nearly half are asymptomatic [4]. These tumours are generally grouped into:

*Peripheral nerve tumours*, which are the most common (70%) mediastinal neurogenic tumours and originate from spinal or proximal intercostal nerve; however, they rarely arise from the vagus, recurrent laryngeal and phrenic nerve [30] (Fig. 29). Schwannomas are the most common (50%) mediastinal neurogenic tumours and frequently affect patients from 20 to 30 years old [30]. They are usually solitary and encapsulated masses, but multiple schwannomas may be associated with neurofibromatosis type 2. The tumour may grow through the adjacent intervertebral foramen and spinal canal to produce a “dumbbell” or “hourglass” configuration. Cystic changes and haemorrhage are more common in schwannomas than in neurofibromas. Neurofibromas are non-encapsulated soft tissue tumours and account for approximately 20% of mediastinal neurogenic tumours [30]. A sudden increase in the size of a previously stable neurofibroma and the presence of neurological symptoms suggests malignant transformation to malignant peripheral nerve sheath tumour. These tumours are closely associated with neurofibromatosis and show more heterogeneous signal intensity and contrast enhancement on MRI.

**Fig. 30:**
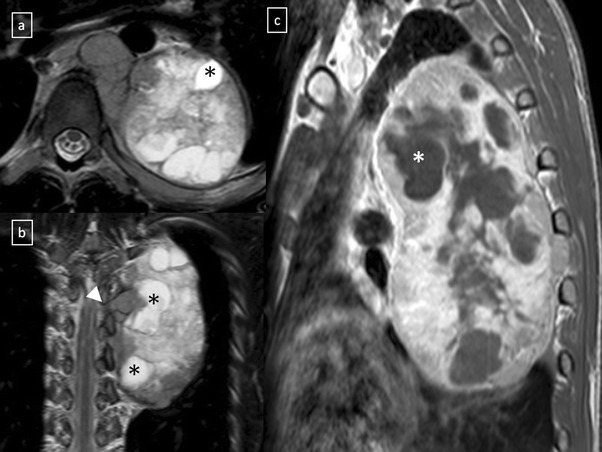
Neuroblastoma in a 20-year-old man. Axial (**a**) and coronal (**b**) T2-weighted MR images, and sagittal (**c**) contrast-enhanced T1-weighted MR image demonstrate an expansive and heterogeneous mass in the left paravertebral space which shows cystic degeneration within (*) as well as an intensive enhancement (in **c**). Note the spinal involvement (*arrowhead* in **b**)

*Sympathetic ganglion tumours*, which comprise 25% of mediastinal neurogenic tumours and arise from neuronal cells rather than from the nerve sheath [30]. Ganglioneuromas are the most benign and differentiated of the autonomic ganglionic tumours. Radiographically, the tumours are well-marginated, occurring along the anterolateral aspect of the spine and spanning three to five vertebrae. The “whorled appearance” is due to curvilinear bands of low signal intensity that reflects collagenous fibrous tissue in the mass on T2-weighted images. Most ganglioneuromas show gradual and heterogeneous contrast enhancement. Ganglioneuroblastomas are the least common type of neurogenic tumour and show intermediate features in cellular maturity between neuroblastoma and ganglioneuroma. Neuroblastomas are highly aggressive and readily metastasising tumours of neuroectodermal origin with a median age at diagnosis of 22 months [30]. They are heterogeneous and non-encapsulated lesions, often exhibiting haemorrhage, necrosis, calcification or cystic degeneration (Fig. 30).

**Fig. 31:**
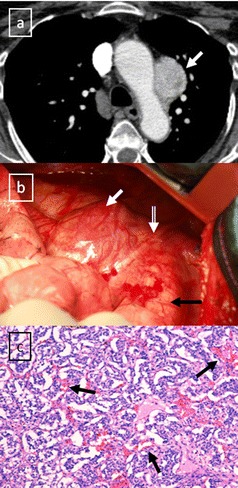
Aortopulmonary paraganglioma in a 52-year-old woman. **a** Enhanced-CT scan shows a markedly enhancing lesion (*arrow*) located adjacent of the arch of aorta. **b** Image during the surgical remove of the lesion. Paraganglioma (*white arrow*); descending aorta (*black arrow*); vagus nervus (*open arrow*). **c** Photomicrograph demonstrated a trabecular pattern of growth and scattered ganglion-like cells surrounded by fibrovascular septa (*arrows*)

*Mediastinal paraganglia.* Paraganglioma is a rare neuroendocrine tumour of chromaffin cell origin. One to two percent of extra-adrenal paragangliomas occur in the thorax [30]. Aortopulmonary paragangliomas are usually asymptomatic, while aortosympathetic paragangliomas (along the sympathetic chain in the posterior mediastinum) occur in symptomatic patients related to the functional activity of the tumour. These masses commonly enhance brightly at enhanced CT (Fig. 31). A characteristic MRI finding of paragangliomas is the presence of multiple curvilinear and punctate signal voids, which reflect high velocity flow in the intratumoral vessels, described as “salt-and-pepper” appearance.

Clinical and radiological features of the most common mediastinal masses are detailed in Table 3.

**Table 3:** Clinical and radiological features of the most common mediastinal masses

	Anatomical location	Imaging features (CT)	Imaging features (MRI)	Manifestations
Lipoma	anterior mediastinum	encapsulated homogeneous fat attenuation (-40 to -120 HU)	homogeneous hyperintensity on T1-WI	asymptomatic (occasionally local compression of surrounding structures)
Liposarcoma	posterior mediastinum	- inhomogeneous fat attenuation	inhomogeneous appearance	symptomatic at presentation
		- irregular areas of soft-tissue appearance		
		- locally aggressive tumours		
Thymolipoma	cardiophrenic angle	- fat-containing lesions (up to 90% fat content)	- homogeneous hyperintensity on T1-WI	- asymptomatic (occasionally local compression of surrounding structures)
		- variable component of soft-tissue elements	- small amounts of solid areas and fibrous septa	- a/w myasthenia gravis, Graves disease and haematological disorders
Bronchogenic cyst	middle/posterior mediastinum	- well-defined homogeneous fluid attenuation (0–20 HU)	- homogeneous hyperintensity on T2-WI	40% symptomatic at presentation
Duplication cyst		- variable composition of fluid if complication or presence of protein or mucoid material	- variable patterns of signal intensity on T1-WI because of variable cyst contents	asymptomatic
Pericardial cyst	cardiophrenic angle			asymptomatic
Neuroenteric cyst	posterior mediastinum	- well-defined homogeneous low-attenuation paravertebral mass	homogeneous hyperintensity on T2-WI	a/w neurofibromatosis, vertebral and rib anomalies or scoliosis
		- enlargement of intervertebral foramina		
Thymic cyst	anterior mediastinum	*CONGENITAL THYMIC CYST:* unilocular well-defined fluid attenuation mass	homogeneous hyperintensity on T2-WI	asymptomatic
		*ACQUIRED THYMIC CYST:* multilocular well-defined fluid attenuation mass with a clearly seen wall	homogeneous hyperintensity on T2-WI with fibrous septa	a/w thymic tumours, after thoracotomy or radiation therapy for HD or as sequelae of inflamatory processes
Mediastinal goitre	anterior mediastinum	- spontaneous hyperattenuation	- heterogeneous appearance on T2-WI	asymptomatic or airway/oesophageal compression
		- inhomogeneous density with cystic areas and calcifications	- relatively hypointensity on T1-WI as compared with normal gland tissue, except foci of haemorrhage and cysts	
		- markedly contrast-enhancement		
Thymic hyperplasia	anterior mediastinum	*TRUE THYMIC HYPERPLASIA:* enlargement of the thymus which remains normally organised	- similar MR signals to those of normal thymus	after chemotherapy, corticosteroid therapy, irradiation or thermal burns
		*LYMPHOID HYPERPLASIA*: normal, enlarged or as a focal thymic mass	- apparent decrease in the signal intensity of the thymus at opposed-phase images in contrast to in-phase images	a/w myasthenia gravis, rheumatoid arthritis, scleroderma, Graves disease
Thymoma	anterior mediastinum	- soft-tissue attenuation	- low signal intensity on T1-WI	- patients older than 40 years-old
		- mild to moderate contrast enhancement	- relatively high signal intensity on T2-WI	- asymptomatic vs pressure-induced symptoms
		- occasionally, focal haemorrhage, necrosis, cyst formation and linear or ring-like calcifications	- complete obliteration of the adjacent fat planes highly suggests mediastinal invasion	- a/w myasthenia gravis (30-50%), hypogammaglobulinaemia (10%) and pure red cells apasia (5%)
		- pleural nodules		
Thymic carcinoma	anterior mediastinum	- ill-defined soft-tissue mass	- similar features on CT	- mean age of 50 years
		- necrotic or cystic component	- absence of tumour capsule	- symptomatic at presentation
		- heterogeneous contrast enhancement		
		- lymphadenopathy and great vessel invasion		
		- 50–65% distant metastases at presentation		
Lymphoma	anterior mediastinum	- homogeneous soft-tissue mass	various signal patterns on T1- and T2-WI	- the most common cause of masses in the paediatric mediastinum
		- mild to moderate contrast enhancement		- HD: bimodal distribution of incidence. Constitutional symptoms
		- cystic and necrotic changes		- TCLL: children and adolescents. Pressure-induced symptoms
		- absence of vascular involvement		- DLBCL: mean age of 30 years
		- mediastinal lymphadenopathy		
		- pleural and pericardial effusions		
Teratoma	anterior mediastinum	- well-defined unilocular or multilocular cystic lesion containing fluid, soft tissue, and fat attenuation. Calcifications may be present	- heterogeneous signal intensity	usually asymptomatic
		- 15% as nonspecific cystic lesion	- fat-fluid levels within the lesion are virtually diagnostic of teratoma	
NTGCT	anterior mediastinum	- heterogeneous ill-circumscribed large mass	heterogeneous signal intensity	- symptomatic young males
		- pericardial and pleural effusion		- tumour markers ß–hCG and AFP
		- involvement of great vessels		
		- distant metastases		
Schwannoma	posterior mediastinum	- markedly convex mass	homogeneous or heterogeneous high signal intensity on T2-WI	- patients from 20 to 30 years old
		- “dumbbell” or “hourglass” configuration		- a/w neurofibromatosis type 2 when multiple
		- cystic, haemorrhage and calcification elements are common		
SGT	posterior mediastinum	- well-defined or ill-defined mass oriented along the anterolateral surface of several vertebrae	heterogeneous high signal intensity on T2-WI	children and young adults
		- “whorled appearance”		
Paraganglioma	APP: along great vessels	hypervascular tumours	“salt and pepper” appearance	APP: asymptomatic patients older than 40 years
	ASP: posterior mediastinum			ASP: younger adults. Half of them present symptoms

*NTGCT* non-teratomatous germ cell tumours, *SGT* sympathetic ganglion tumours, *AFP* alpha-fetoprotein, *ß–hCG* beta human chorionic gonadotropin, *HD* Hodgkin disease, *TCLL* T-cell lymphoblastic lymphoma, *DLBCL* diffuse large B-cell lymphoma, *APP* aortopulmonary paraganglioma, *ASP* aortosympathetic paraganglioma, *a/w* association with, *WI* weighted imaging

### Uncommon mediastinal masses

**Fig. 32:**
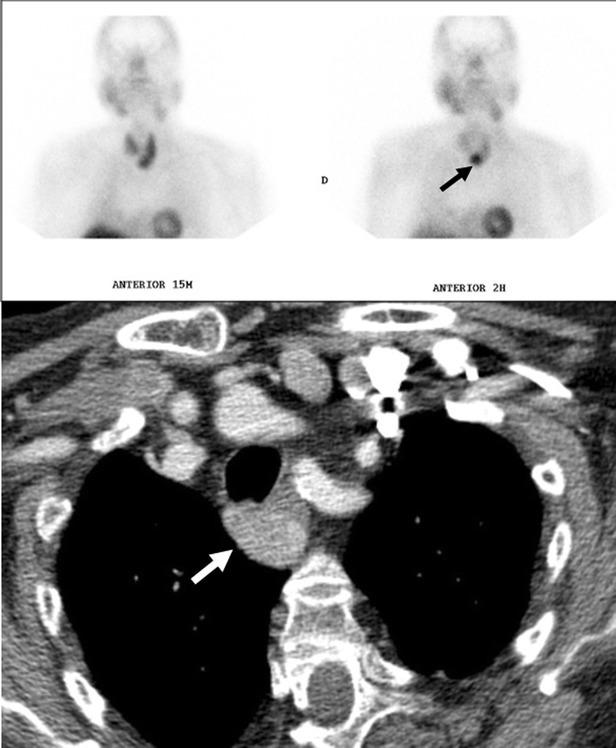
Parathyroid adenoma in a 66-year-old man with hypercalcaemia, hypophosphataemia and elevated PTH values. Tc-99m MIBI scan shows a focus of hyperactivity (*black arrow*) adjacent to the lower pole of left thryoid lobule. Enhanced-CT scan shows a superior mediastinal enhanced mass (*white arrow*)

**Fig. 33:**
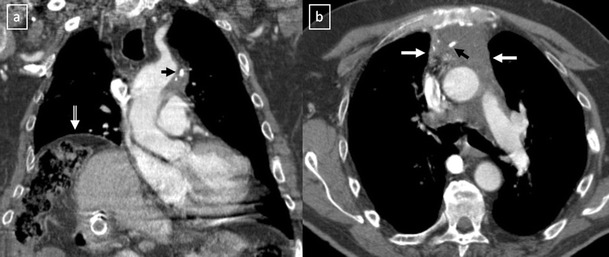
Idiopathic fibrosing mediastinitis in a 64-year-old man. **a** Coronal multiplanar reconstruction and axial **b** constrast-enhanced CT scan show an infiltrating soft tissue mass (*white arrows*) in mediastinum encasing major vessels. Punctate calcifications are observed (*black arrows*) as well as an elevation of the right hemidiaphragm (*open arrow*) secondary to the phrenic nerve involvement

**Fig. 34:**
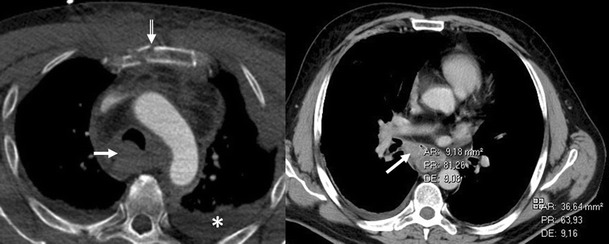
**a** Contrast-enhanced CT scan of a man who had suffered a traffic accident. An infiltrative mediastinal haematoma is identified with subtle areas of high CT-attenuation values (*arrow*). Bilateral pleural effusion (*) and a sternum fracture (*open arrow*) are also observed. **b** Iatrogenic mediastinal haematoma (*arrow*) in a 64-year-old man secondary to bronchoscopy with transtracheal biopsy. Note the high attenuation value of the lesion compared with muscular tissue

*Parathyroid adenomas* may be seen in ectopic locations, the mediastinum being the most commonly site. High-resolution ultrasonography (US) is recognised as a tool for detecting cervical parathyroid lesions. As it enlarges, an abnormal gland appears as a hypoechoic, and often anechoic, lesion, often posterior in location to the thyroid. As the gland enlarges, it can develop lobularity and foci of echogenicity. Colour Doppler assessment of parathyroid lesions is a useful integration of grey-scale US and may be helpful in featuring parathyroid lesions. The colour Doppler patterns termed “parenchymal” (pattern IV, internal flow) and “vascular pole” (pattern II, focal peripheral flow) are typical of parathyroid lesions [31]. The different colour Doppler US patterns seem to be influenced by many factors as the location of the gland and the degree of vascularity. These tumours tend to be small and may contain calcifications at CT. Technetium-99 Sestamibi SPECT scans are more effective for their diagnosis (Fig. 32). *Fibrosing mediastinitis* is a dense fibrosis which progressively encases and eventually obliterates the lumen of the mediastinal vessels and airways (Fig. 33). *Haematoma.* High attenuation of haematomas can be observed on unenhanced CT scans during the first 72 h (Fig. 34). When the hematoma ages its attenuation decreases at CT in a centripetal fashion. *Haemangiomas* in the mediastinum are rare and the may be associated with Rendu-Osler syndrome. *Sarcomas* other than vascular or neural origin, including fibrosarcomas, osteosarcomas and chondrosarcomas, are also very uncommon. *Extramedullary haematopoiesis* in posterior mediastinum is another entity to take into account.

## Follow-up

In assessment of mediastinal disease, cross-sectional imaging techniques allow excellent visualisation of the mediastinum. CT is generally the first-choice modality of diagnostic imaging. MRI plays an increasing role in this disease due to the existence of new available MR techniques on mediastinum imaging.

On each of CT and MR scanning, the size of tumour, contour, perimeter of capsule, septum, haemorrhage, necrotic or cystic component, homogeneity within tumour, presence of mediastinal lymphadenopathy, pleural effusion and great vessel invasion are assessed. In addition, presence of calcification are assessed on CT and signal intensities of the tumour are assessed on MRI.

Imaging plays an essential role in the diagnosis, staging and follow-up of mediastinal disease. Complete resection is the mainstay of treatment in many mediastinal tumours and the ability to accomplish a complete resection appears to be the most important prognostic factor. Currently, CT is the modality most commonly used for follow-up after treatment. The goal of follow-up is to detect recurrence as early as possible. CT findings may serve as predictors of tumour invasiveness and of postoperative recurrence or metastases.

In Table 4 we summarise some teaching points and imaging pitfalls for the diagnostic approach to mediastinal masses before and after treatment.

**Table 4:** Teaching points and imaging pitfalls for the diagnostic approach to mediastinal masses before and after treatment

• CT is accurate in distinguishing mediastinal masses which usually differ in their appearance and the pattern of metastatic spread, both of which are readily detected by chest CT
• Pericardial fat pads and lipomatosis are correctly interpreted as normal findings rather than possible pathological lesions
• When lipoma and liposarcoma are situated in the cardiophrenic space, the imaging findings are very similar to those of Morgagni hernia
• MRI more accurately distinguishes between cystic and solid lesions than CT
• Soft-tissue components associated with cystic lesions can be related to a malignant process (e.g. soft-tissue nodules in a cystic anterior mediastinal lesion suggest that the lesion is a cystic thymoma rather than a congenital cyst)
• Non-neoplastic thymic enlargement must not be confused with thymoma. The normal thymus in young children and the hyperplastic thymus may mimic a mass
• When differentiation between non-neoplastic thymic enlargement and thymoma cannot be achieved at CT or conventional MRI, chemical-shift MRI with in-phase and out-of-phase gradient-echo sequences can be helpful
• Thymoma rarely manifests with lymphadenopathy, pleural effusions, or extrathoracic metastases
• The role of imaging is to initially diagnose and properly stage thymoma, with emphasis on the detection of local invasion and distant spread of disease, to identify candidates for preoperative neoadjuvant therapy
• Late recurrence in thymoma is not uncommon. Imaging of treated patients is directed at identifying resectable recurrent disease, since patients with completely resected recurrent disease have similar outcomes as those without recurrence [23]
• Findings associated with significantly more frequent recurrence and metastases of thymic tumours include lobulated or irregular contour, oval shape, mediastinal fat invasion or great vessel invasion and pleural seeding
• Mediastinal lymphadenopathy, pleural effusions, and pulmonary metastases are characteristic of thymic carcinoma or non-teratomatous germ cell tumour
• Lymphoproliferative disorders typically present pleural effusions, pericardial fluid, and mediastinal lymphadenopathy in many cases
• Heterogeneous appearance (due to necrosis, cystic change, or haemorrhage) is typical of thymic carcinoma, lymphoma, sympathetic ganglion tumour, peripheral nerve tumour and non-teratomatous germ cell tumour. It can be seen in about one-third of thymomas
• A cystic anterior mediastinal mass with intrinsic fat attenuation typically represents a mature teratoma

## Conclusion

Tumours of the mediastinum represent a wide diversity of disease state. The location and composition of a mass is critical to narrowing the differential diagnosis. The clinical spectrum of mediastinal masses can range from being asymptomatic to producing compressive symptoms. Although many of these masses have similar imaging appearances, clinical history, anatomical position and certain details seen at CT and MRI imaging allow correct diagnosis in many cases.
